# Atrichia With Papular Lesions Confirmed via Genetic Testing: A Case Report

**DOI:** 10.7759/cureus.32562

**Published:** 2022-12-15

**Authors:** Julie Boisen, Jade Lewis, Sophia J Hendrick

**Affiliations:** 1 Dermatology, Baylor Scott & White Health, Temple, USA; 2 General Medicine, United States Navy, Jacksonville, USA

**Keywords:** pediatric alopecia, hairless gene, pediatric hair loss, alopecia, atrichia with papular lesions

## Abstract

Atrichia with papular lesions (APL) is a rare form of alopecia characterized by the diffuse, complete, irreversible loss of hair shortly after birth and the presence of diffuse keratotic papules and milia-like cysts. Multiple hairless gene (*HR*) mutations on the zinc finger domain of chromosome 8p12 have been associated with this disorder. We present the case of a 5-year-old girl with classic clinical findings of APL, with a diagnosis confirmed via genetic testing.

## Introduction

Atrichia with papular lesions (APL) is a rare form of alopecia characterized by the diffuse, complete, irreversible loss of hair shortly after birth with the presence of diffuse keratotic papules and milia-like cysts. The diagnosis of APL requires a detailed clinical and family history, as well as DNA sampling to identify a hairless gene (*HR*) mutation [1]. Over 30 mutations of the hairless gene (*HR*) on chromosome 8p12 have been associated with APL. Though the majority of cases are associated with consanguinity, sporadic heterozygous mutations have been identified as well [2].

## Case presentation

**Figure 1:**
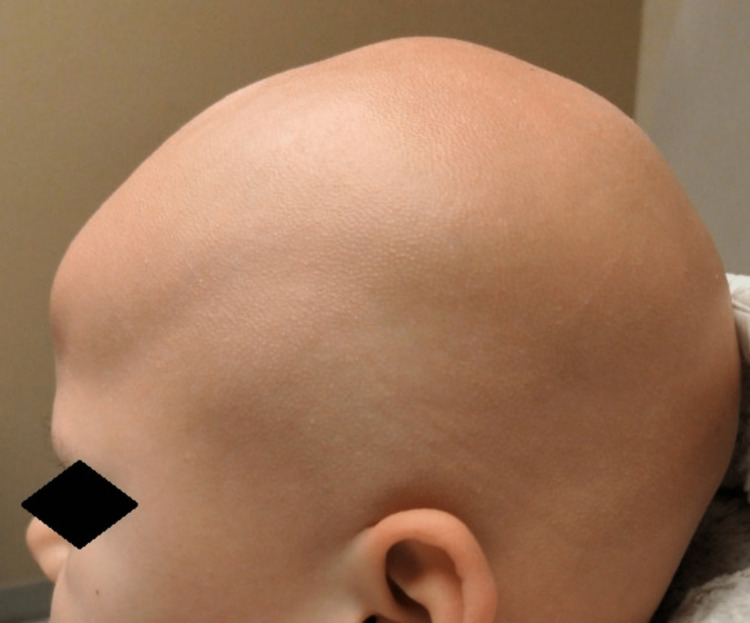
The left side of the scalp showing absence of hair and papular lesions. The left eyebrow is sparse.

**Figure 2:**
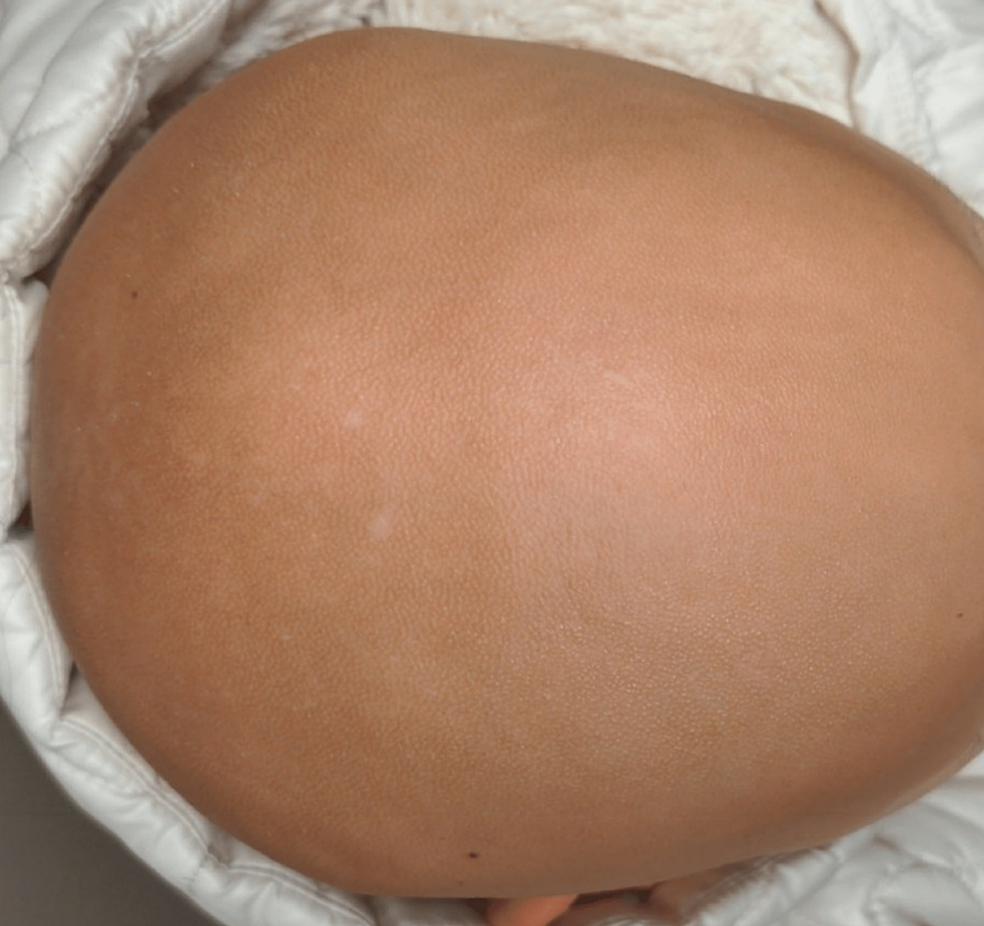
Top view of the scalp showing absence of hair and papular lesions.

A 5-year-old girl presented for congenital absence of the majority of scalp hair and hair loss. She had a small amount of black scalp hair in patches at birth. Between 6 and 12 months of age, she lost the majority of her hair with no subsequent regrowth. A physical exam revealed one terminal hair on the vertex scalp, but there was otherwise a complete absence of scalp hair. Many skin-colored to white 1-2 mm papules were seen on the scalp. The eyebrows and eyelashes were sparse with minimal hair growth (Figures 1, 2). She had previously undergone genetic testing, which confirmed a diagnosis of APL with a biallelic mutation in the *HR* gene with likely pathogenic variants, including c.3515G>A and p.W1172X. Her mother also had genetic testing and was heterozygous for the *HR* gene; however, she had normal hair density. It was discovered that consanguinity was present, as the patient’s biological father was her mother’s maternal cousin. The patient had a history of global developmental delay without a known specific causative genetic mutation. As the diagnosis of APL had been confirmed via genetic testing, scalp biopsy was deferred. The patient was offered treatment with topical tretinoin 0.05% cream to reduce milia-like lesions. The patient followed up via telephone, and her mother reported trialing the tretinoin cream for two months, but it was discontinued due to skin irritation.

 

## Discussion

APL is a rare, autosomal recessive genodermatosis associated with diffuse hair loss that typically occurs within the first year of life [3]. Though this disease can mimic alopecia universalis congenita (AUC), the formation of keratin-filled cysts in a diffuse distribution in the first year of life is pathognomonic of APL and is usually absent in AUC [2,3]. Eruptive milia can occur spontaneously and in association with other autosomal dominant syndromes or genodermatoses [4].

Alopecia due to vitamin D-dependent rickets type IIA (VDDR IIA) has many clinical and histologic similarities to APL and AUC. Patients are born with normal hair distribution and experience hair loss within 12 months and may have milia-like cysts. On histology, the three entities are strikingly similar with the main findings, including empty infundibula; absence of the lower two-thirds of the hair follicles, often replaced by vertically oriented irregular epithelial structures or epithelial cysts; irregular epithelial structures often with small cysts in the middle and lower dermis; and small, medium, and large keratinizing epithelial cysts at all levels of the dermis [5]. Cysts are often present histologically, even if they are not clinically apparent. Genetic testing can help guide the diagnosis of APL, AUC, and VDDR IIA by identifying the different causative mutations.

Multiple hairless gene (*HR*) mutations on the zinc finger domain of chromosome 8p12 have been associated with APL and AUC. These mutations are thought to cause reduced *HR* function, resulting in an increase of premature intrafollicular apoptosis in the infundibular and isthmic portion of the outer root sheath (ORS) cells before the first adult hair cycle [6,7]. VDDR IIA is caused by mutations of the vitamin D receptor (VDR) gene on chromosome 12.

Patients with APL usually meet developmental milestones and have no evidence of delayed bone growth, seizures, hearing loss, or additional defects in ectodermal structures, unlike vitamin D-dependent rickets or ectodermal dysplasia. APL is likely underdiagnosed due to misdiagnosis as AUC and failure to perform confirmatory testing, especially in patients who lack a history of consanguinity [2,3]. When possible, confirmatory genetic testing, along with clinical findings, is the gold standard for diagnosis. Although histological sampling of the scalp is not required, it should be utilized to confirm APL when genetic testing is not available [2]. This helps to avoid unnecessary treatment with systemic steroids or disease-modifying antirheumatic drugs (DMARDs), which are not efficacious in treating APL. Topical tretinoin has been used in the treatment of spontaneous eruptive milia, but the results are variable [4].

## Conclusions

We present the case of a 5-year-old girl with classic clinical findings of APL, with a confirmed mutation of the hairless gene (*HR*). Dermatologists should recognize the clinical features of APL and similar disease processes such as AUC and VDDR IIA and refer for genetic testing for definitive diagnosis as distinguishing these entities via histology may not be possible.
